# Intrinsic sensory disinhibition contributes to intrusive re-experiencing in combat veterans

**DOI:** 10.1038/s41598-020-57963-2

**Published:** 2020-01-22

**Authors:** Kevin J. Clancy, Alejandro Albizu, Norman B. Schmidt, Wen Li

**Affiliations:** Department of Psychology, Florida State University, Tallahassee, FL USA

**Keywords:** Neuroscience, Psychology

## Abstract

Intrusive re-experiencing of traumatic events is a hallmark symptom of posttraumatic stress disorder, characterized by rich and vivid sensory details as reported in “flashbacks”. While prevailing models of trauma intrusions focus on dysregulated emotional processes, we hypothesize that a deficiency in intrinsic sensory inhibition could drive overactivation of sensory representations of trauma memories, precipitating sensory-rich intrusions. In a sample of combat veterans, we examined resting-state alpha (8–12 Hz) oscillatory activity (in both power and posterior→frontal connectivity), given its role in sensory cortical inhibition, in association with intrusive re-experiencing symptoms. Veterans further participated in an odor task (including both combat and non-combat odors) to assess olfactory trauma memory and emotional response. We observed an association between intrusive re-experiencing symptoms and attenuated resting-state posterior→frontal alpha connectivity, which were both correlated with olfactory trauma memory. Importantly, olfactory trauma memory was identified as a mediator of the relationship between alpha connectivity and intrusive re-experiencing, suggesting that deficits in intrinsic sensory inhibition contributed to intrusive re-experiencing of trauma via heightened trauma memory. Therefore, by permitting unfiltered sensory cues to enter information processing and activate sensory representations of trauma, sensory disinhibition can constitute a sensory mechanism of intrusive re-experiencing in trauma-exposed individuals.

## Introduction

Intrusive re-experiencing is a hallmark symptom of posttraumatic stress disorder (PTSD), characterized by the involuntary reactivation or reliving of trauma memories^1^. To date, predominant models of PTSD have attributed these intrusive trauma memories to heightened emotion processing including excessive threat detection, exaggerated threat appraisal, and dysfunctional emotion regulation^2–4^. These dysfunctions in PTSD include deficient top-down, volitional suppression of intrusions, as the prefrontal cortex fails to inhibit the response of the sensory cortex and hippocampus to trauma cues^5–7^, as well as exaggerated bottom-up processing of threat cues through the rapid “tagging” of threatening stimuli by midbrain and limbic structures^8–10^. Complementing this deficiency in top-down prefrontal voluntary inhibition and exaggerated bottom-up limbic threat detection, the role of sensory cortical inhibition of incoming sensory cues in intrusive re-experiencing of trauma has not been fully examined.

This sensory mechanism draws support from the dual representation theory of PTSD, highlighting a sensory-bound representation system of threat memory (“S-memory”) that can be activated by basic sensory inputs and trigger the re-experiencing of traumatic events^11,12^. This sensory mechanism could account for intrusions that are dominated by vivid, low-level sensory fragments of trauma and readily triggered by simple sensory cues^13–16^. Furthermore, the bottom-up, intrinsic nature of this sensory mechanism could be particularly relevant to the spontaneous, involuntary quality of intrusions.

Aberrant low-level sensory processing has been reliably observed in PTSD, implicating sensory disinhibition in the pathology of the disorder^17^. Patients with PTSD often report feeling ‘flooded’ by everyday sensory stimuli, such as background noises that others would not notice. These reports are corroborated by systematic sensory profiling, which reveals sensory filtering/gating deficits and sensory hypersensitivity in these patients^18,19^. Electrophysiological data further associate PTSD with impaired sensory gating, evinced by poor repetition suppression to repeated auditory (“double-click”) stimuli (i.e., reduced P50 response), and sensory cortical hyperactivity, evinced by exaggerated sensory evoked brain potentials and mismatch negativity in response to simple, neutral stimuli^18,20–22^. According to the dual representation theory, such sensory disinhibition (as deficient sensory gating and hyperactivity) could allow unfiltered sensory inputs to reach the sensory cortex to over-activate sensory representations of trauma memory (“S-memory”), resulting in sensory-rich intrusions.

A key neural mechanism underlying sensory inhibition involves alpha-frequency (8–12 Hz) oscillations, which dominate neural synchrony in the awake restful state^23–26^. Specifically, alpha oscillatory activity exerts inhibitory influences on sensory cortical neuronal firing and excitation^27–29^. Furthermore, via long-range, posterior→frontal projections, alpha oscillations mediate inhibitory bottom-up information flow from the sensory cortex to frontal regions to influence global neural activity^30–33^. This long-range alpha connectivity plays a critical role in gating the entry of sensory input into downstream processing and, eventually, conscious awareness, thereby regulating perception, imagery, and working memory^34–38^. As we have recently demonstrated, alpha activity is suppressed in patients with PTSD, including both local alpha power and posterior→frontal alpha connectivity^17^. This is consistent with a growing body of literature identifying aberrant oscillatory activity, specifically, reduced alpha oscillations, in PTSD that spans posterior and frontal cortical regions^39–44^. We therefore hypothesize that deficient alpha activity could compromise sensory inhibition such that unfiltered sensory input would flood information processing, activating stored memory representations and eliciting vivid imagery and perception, akin to intrusive re-experiencing of trauma.

**Figure 1:**
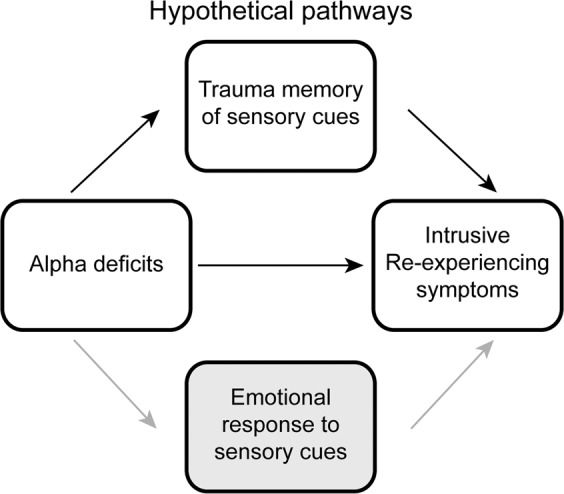
A sensory model for intrusive re-experiencing symptoms, testing the hypothesis that deficient inhibition of sensory information increases intrusive re-experiencing symptoms by increasing trauma memory recall. Emotional response to sensory cues was considered as a competing mediator in this model.

To test this hypothesis, we recruited a sample of combat-exposed veterans (*N* = 86) and examined their intrusive re-experiencing symptoms in relation to alpha activity, including alpha power and alpha posterior→ frontal connectivity (using Granger causality analysis)^45^. We focused on intrinsic, resting-state (as opposed to task-positive or cue-related) alpha activity to elucidate the spontaneous, involuntary nature of intrusions. In a subsample of veterans (*N* = 35), we further tapped into sensory-based activation of autobiographical memory of combat experience. We chose olfactory (combat and non-combat) cues to activate olfactory trauma memory given that odors are known to activate vivid, sensory-rich memories in a Proustian manner^46,47^ and strongly trigger trauma memories in PTSD^48–50^. As illustrated in Fig. 1, we tested our hypothesis in a mediation model, where intrinsic alpha deficits contributed to intrusive re-experiencing through overactivation of sensory-based trauma memory. To disambiguate this memory mechanism from the potentially competing mechanism of exaggerated threat processing (as prominently implicated in PTSD)^2^, we also assessed emotional response to odors to pit against olfactory trauma memory in relation to alpha attenuation and intrusive re-experiencing.

## Methods

### Participants

Ninety-two combat-exposed veterans participated in the study after providing informed consent approved by the Florida State University Institutional Review Board and the Department of Defense Human Research Protection Official’s Review. The experiment was performed in accordance with approved relevant guidelines and regulations. Participants had no history of severe neurological disorders or traumatic brain injury, no current or past psychotic spectrum or bipolar disorders, and no current substance dependence or abuse of opioids, stimulants, or cocaine. Traumatic brain injury was assessed through an eligibility screening including (1) history of hospitalization or emergency care following a head or neck injury, (2) loss of consciousness from an accident or injury, (3) head or neck injury from a vehicular accident, and 4) head or neck injury from a fight or a fall. Information was further validated during a clinical interview (detailed below). Six participants were excluded due to excessive EEG artefact or failure to follow instructions, resulting in a final sample of eighty-six veterans (mean age: 45.9 ± 12.6 years; 10 females).

Participants here belonged to a large project. A subsample of thirty-five veterans (mean age: 42.6 ± 13.4 years; 4 females) were recruited, following the first wave of recruitment, to undertake an additional olfactory task (see below for details). Additional demographics are provided in Table 1.

**Table 1:** Participant Demographics (*n* = 86). PCL = Posttraumatic Stress Disorder Checklist; BDI = Beck Depression Inventory.

	Full Sample	Olfactory Subsample
Age (years)	45.9 ± 12.6	42.6 ± 13.4
Gender (female/male)	10/76	4/31
Medication Use (%)	28 (20.9%)	10 (28.6%)
PTSD (primary, present)	25 (29.1%)	8 (22.9%)
w/ Comorbid diagnosis	15 (60%)	5 (62.5%)
PCL Total Score	40.4 ± 21.9	33.8 ± 15.6
PCL Intrusions Cluster	7.7 ± 5.5	6.7 ± 5.0
BDI	21.0 ± 12.8	15.1 ± 9.4

### Clinical Assessment

Diagnoses were assessed using the Structured Clinical Interview for DSM-V^1^, among which 25 (29.1%) met DSM-V criteria for current PTSD. 8 of the 35 (22.9%) participants in the olfactory subsample met diagnostic criteria for PTSD, comparable to the rate in the total sample. Participants also completed the PTSD Checklist (PCL)^51^, from which we summed the responses to Items 1–5 that assess symptoms of intrusions to generate a total score of intrusive re-experiencing symptom severity (range: 0–20). Additionally, participants completed the Beck Depression Inventory (BDI-II)^52^ to index general depression symptoms additionally associated with intrusions. Scores on these measures are provided in Table 1.

### EEG acquisition and analyses

EEG data were recorded from a 96-channel BrainProducts actiChamp system (1000 Hz sampling rate, 0.05–200 Hz online bandpass filter, referenced to the FCz channel) for 2 minutes, with eyes fixated on the central crosshair of a computer screen. Given the high sampling rate (e.g., 1000 Hz), resting-state EEG recordings of 2 minutes provide sufficient power and reliability for spectral analysis and have been commonly used by our lab and others^17,53–56^. Moreover, given the prevalence of behavioral symptoms among the veterans (e.g., hyperarousal, difficulty in sustaining attention) that could disrupt the “resting state” as we instructed (i.e., maintaining alert restfulness with eyes fixated on the crosshair), such 2-minute recordings would be consistently tolerable to all participants. Electro-oculogram (EOG) was recorded using four electrodes with vertical and horizontal bipolar derivations. EEG/EOG data were downsampled to 250 Hz, high-pass (1 Hz) and notch (60 Hz) filtered, and re-referenced to the average of all EEG channels. We applied the *Fully Automated Statistical Thresholding for EEG artifact Rejection* (FASTER) algorithm for artifact detection and rejection^57^. FASTER uses a *z*-score threshold of ± 3 to detect and correct physiological (i.e., heartbeat, eyeblinks, movement) and non-physiological (i.e., electrode “pop-off”) artifacts within single channels, individual epochs, independent components, and within-epoch channels. Additional details are provided in the Supplemental Methods.

### Power analyses

**Figure 2:**
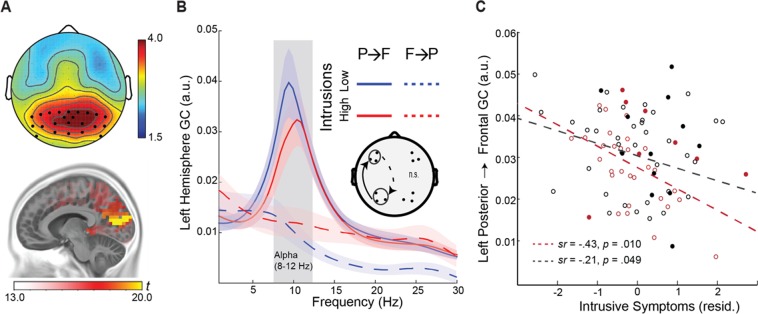
Attenuated left-hemisphere alpha connectivity was related to greater intrusion symptoms. (**A**) Topography of alpha power, maximally distributed over occipitoparietal electrodes. Intracranial sources of alpha power were localized to the striate/extrastriate visual cortices (e.g., maximum in the cuneus; x, y, z = −10, −80, 10). (**B**) Spectra of left-hemisphere Granger causality for participants with high vs. low intrusive re-experiencing symptoms (median split), demonstrating reduced alpha (8–12 Hz) posterior→frontal connectivity in high-intrusion participants. The opposite, frontal→posterior GC was minimal and equivalent for the two groups. Shaded ribbons = standard error of the mean (SEM). (**C**) Negative correlation between alpha connectivity and intrusive re-experiencing symptoms, controlling for depression symptoms (BDI scores), in the olfactory (red) and full (black) samples. Solid circles reflect participants with a diagnosis of PTSD. P→F = posterior→frontal; F→P = frontal→posterior.

EEG oscillation power was computed for individual channels for each epoch (1-sec) using the multitaper spectral estimation technique^58^. Alpha (8–12 Hz) power was normalized by the mean power for the global spectrum (1–50 Hz) within each epoch, and averaged across occipito-parietal electrodes, where alpha is maximally distributed^23,24,29^ (Fig. 2A). Figure 2A further illustrates the intracranial alpha source, localized to the striate/extrastriate visual cortices (e.g., maximum in the cuneus; x, y, z = −10, −80, 10) based on the exact Low-Resolution Electromagnetic Tomography (eLORETA)^59^, consistent with prior literature^59–61^.

### Directed alpha-frequency connectivity (Granger causality) analyses

Alpha-frequency Granger causality (GC) analysis^45,62^ was performed to assess posterior→frontal causal connectivity in the alpha band. Following transformation into reference-free, current source density data (CSD) using the surface Laplacian algorithm^63–65^, EEG data from ipsilateral posterior-frontal pairs were submitted to bivariate autoregressive (AR) modeling, from which Granger causality spectra were derived^45,66^. A model order of 20 (80 ms in time for a sampling rate of 250 Hz) was chosen in a two-step process: (1) Akaike Information Criterion (AIC) and (2) comparing the spectral estimates obtained by the AR model and that by the Fourier based method for data pooled across all subjects^65^. Ipsilateral posterior-frontal electrode pairs were selected *a priori* based on previous studies^17,30^. Akin to the dominance of the posterior→frontal directed propagation of alpha oscillations at rest^32,33,67^, minimal frontal→posterior alpha connectivity was observed (Fig. 2B).

### Odor task

The subsample of thirty-five veterans completed an odor task whereby six combat and six non-combat odors contained in amber glass bottles were presented. Combat odors included two variations of spent gunpowder and burning rubber, as well as the smell of burning flesh and of body decay (ScentAir™, NC). Non-combat odors included neutral (or mildly pleasant) chemicals: cleaning fluid (ScentAir™, NC), acetophenone, eugenol, cedrol, alpha-ionone, and citronellol (Fisher Scientific, NH). Both combat and non-combat odors were rated to be of equal, moderate intensity [mean (SD) = combat: 52.2 (12.7) versus non-combat: 48.7 (11.8); *t*_1,34_ = 1.65, *p* = 0.11].

To assess the level of trauma memory activation by sensory cues, we measured the degree to which the odors were recognized as being experienced during the veterans’ combat deployment. To disambiguate memory and emotion processes, we also assessed the level of emotional responses elicited by the odors. Specifically, on each odor presentation (randomized across the 12 odors), veteran participants were asked to rate on a visual analog scale (VAS; 0–100) how strongly the odor was associated with and how frequently it was experienced during their combat deployment. These two ratings were averaged into an olfactory trauma memory score. Participants were also asked to rate how strongly the odor elicited distress, disgust, and anxious arousal, which were averaged into an olfactory emotional response score. The order of these ratings was randomized across odors. Paired samples *t*-tests revealed that combat odors, relative to non-combat odors, had higher trauma memory scores [mean (SD) = combat odors: 32.83 (14.81) versus non-combat odors: 25.31 (13.85); *t*_1, 34_ = 4.21, *p* < 0.001], and higher emotional response scores [combat odors: 43.46 (15.76) versus non-combat odors: 30.04 (14.99); *t*_1, 34_ = 7.36, *p* < 0.001], confirming their distinct odor categories. Finally, trauma memory and emotional response scores were marginally correlated (combat odor: *r* = 0.32, *p* = 0.063; non-combat odor: *r* = 0.33, *p* = 0.051], indicating that they reflected related but distinct constructs.

### Statistical Analyses

Multiple regression analyses were performed to examine associations between alpha activity (power and GC) and intrusive re-experiencing symptoms, with BDI scores entered as covariates to isolate trauma-specific intrusions from comorbid depressive symptoms. Significant correlations between alpha activity (power or GC) and intrusive symptoms would then motivate omnibus repeated measures analyses of covariance (rANCOVAs) to examine the relation between these variables and olfactory trauma memory and emotional response. Specifically, omnibus rANCOVAs were performed on the olfactory scores with Category (combat vs. non-combat) and Response (combat memory vs. emotional response) as independent variables and alpha activity (power or GC value) as a covariate. “Response” was entered as an independent variable in the ANCOVA to pit olfactory trauma memory against emotional response, thereby isolating specific memory effects. In addition, a similar ANCOVA was conducted with the severity of intrusive re-experiencing symptoms (based on the PCL) as a covariate to examine its association with olfactory trauma memory and emotional response. For thoroughness and mitigation of Type I errors, we started with an omnibus ANCOVA for each key DV, where significant or trending interactions were followed systematically by specific simple tests. All specific tests pertinent to the hypotheses were then further controlled for multiple comparisons using the false discovery rate (FDR) criterion (FDR *p* < 0.05).

Provided that the above variables were correlated with each other, mediation analyses were conducted to test the hypothesis of trauma memory or emotional response mediating the association between alpha activity and intrusive re-experiencing symptoms. The PROCESS macro for SPSS^68^ was used to estimate 5,000 bias-corrected bootstrap samples, from which a 95% confidence interval (CI) was created to test the indirect effect of alpha activity on re-experiencing symptoms through olfactory processes. Again, depression symptoms (BDI scores) were entered as covariates to control for effects of comorbid depression.

## Results

### No effect of alpha power on intrusive re-experiencing

A multiple regression analysis revealed no association between posterior alpha power and intrusive symptoms, after controlling for depression symptoms (*p* = 0.640). Given this null effect, the follow-up rANCOVAs were not justified and thus not reported here. For reference, we presented results of the rANCOVAs with alpha power in the supplemental material.

### Attenuated alpha connectivity associated with greater intrusive re-experiencing

Multiple regression analyses revealed a negative association between alpha GC and intrusive re-experiencing in the left hemisphere (*sr* = −0.43, *p* = 0.010, FDR corrected *p* < 0.05), albeit not in the right hemisphere (*sr* = −0.09, *p* = 0.629), after controlling for depression symptoms (Fig. 2). That is, veterans with attenuated left-hemispheric alpha connectivity showed greater intrusive re-experiencing symptoms. Notably, this pattern of results was reproduced in the full sample of 86 veterans (left hemisphere: *sr* = −0.21, *p* = 0.025 one tailed, FDR corrected *p* < 0.05; right hemisphere: *sr* = −0.01, *p* = 0.371), highlighting the generalizability of this relationship. Sensitivity analyses confirmed the specificity of this association to intrusive symptoms, as other PTSD symptom clusters were not associated with alpha connectivity (*p*’s > 0.424). With this association between alpha connectivity and intrusion symptoms, we conducted the following ANCOVAs, limited to the left hemisphere that exhibited significant effects.

### Olfactory trauma memory associated with greater intrusive re-experiencing symptoms

**Figure 3:**
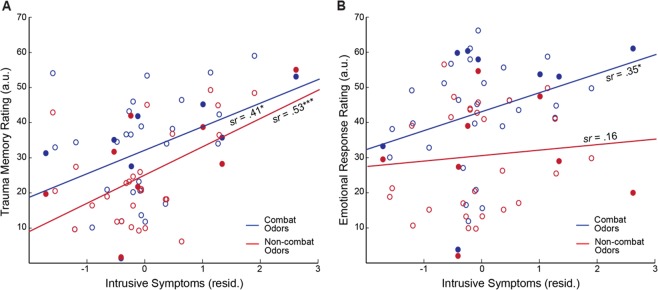
Olfactory responses were associated with intrusion symptoms. (**A**) Greater emotional response to combat (blue), but not non-combat (red), odors was associated with greater intrusive re-experiencing symptoms (controlling for depression). (**B**) Greater trauma memory for both combat (blue) and non-combat (red) odors was associated with greater intrusive re-experiencing symptoms. Solid circles reflect participants with a diagnosis of PTSD. *p < 0.05; **p < 0.01; ***p < 0.005; ^†^*p* < 0.1.

An omnibus rANCOVA of Category (combat vs. non-combat) by Response (memory vs. emotion) by intrusive re-experiencing symptoms on olfactory scores revealed a trending 3-way interaction of Category by Response by intrusive-re-experiencing (*F*_1,33_ = 3.25, *p* = 0.080, *η*_p_² = 0.09). We then broke down the interaction by Response by running two separate follow-up rANCOVAs (Category-by-intrusive re-experiencing) for olfactory trauma memory and olfactory emotion response, separately. For olfactory trauma memory scores, the follow-up rANCOVA showed a main effect of intrusive re-experiencing (*F*_1, 33_ = 11.22, *p* = 0.002, *η*_p_² = 0.25; *sr* = 0.50, FDR corrected *p* < 0.05), but no Category by intrusive re-experiencing interaction (*p* = 0.529). That is, as illustrated in Fig. 3A, trauma memory for both combat (*sr* = 0.41, *p* = 0.013, FDR corrected *p* < 0.05) and non-combat (*sr* = 0.53, *p* = 0.001, FDR corrected *p* < 0.05) odors was significantly associated with intrusive symptoms. A similar rANCOVA was conducted for olfactory emotional response scores, yielding no main effect of intrusive re-experiencing symptoms (*p* = 0.12), and a trending effect of Category X intrusive re-experiencing (*F*_1, 33_ = 2.88, *p* = 0.099, *η*_p_² = 0.08): intrusive symptoms were correlated with emotional response to combat odors (*sr* = 0.35, *p* = 0.041, albeit not FDR corrected) but not with non-combat odors (*sr* = 0.16, *p* = 0.355; Fig. 3B). Therefore, there was an association between intrusive symptoms and general trauma memory (for both combat and non-combat cues) as well as emotional response to combat (but not non-combat) cues.

### Olfactory trauma memory associated with decreased alpha connectivity

**Figure 4:**
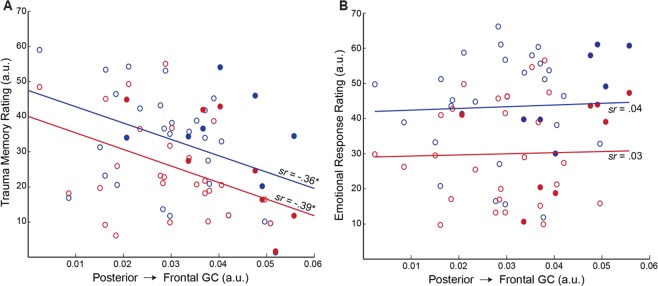
Attenuated alpha connectivity was related to heightened trauma memory for olfactory cues. (**A**) Weakened left-hemisphere posterior→frontal Granger causality was associated with greater trauma memory for both combat (blue) and non-combat (red) odors. (**B**) Left-hemisphere posterior→frontal Granger causality demonstrated no association with emotional response to neither combat nor non-combat odors. Solid circles reflect participants with a diagnosis of PTSD. *p < 0.05.

An omnibus rANCOVA (Category by Response by alpha GC) on olfactory ratings revealed a significant interaction between Response and alpha GC (*F*_1,33_ = 5.19, *p = *0.029*, η*_p_² = 0.14), such that reduced alpha connectivity was associated with greater olfactory trauma memory (*sr* = −0.40, *p* = 0.018, FDR corrected *p* < 0.05; Fig. 4A) but not olfactory emotional response (*p* = 0.839; Fig. 4B). There was no Category effect or Category-by-Response interaction (*p*’s > 0.929), suggesting that the association of trauma memory with alpha connectivity spanned across combat (*sr* = −0.36, *p* = 0.035) and non-combat (*sr* = −0.39, *p* = 0.023) odors (Fig. 4A). Therefore, veterans with reduced alpha posterior→frontal connectivity also exhibited heightened olfactory trauma memory (to both trauma and non-trauma odors) but not exaggerated emotional response.

### Olfactory trauma memory mediating the association between alpha connectivity and intrusive re-experiencing

**Figure 5:**
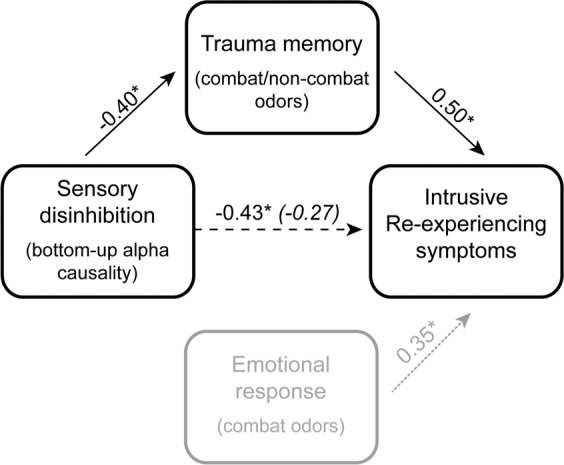
Mediation: Trauma memory for olfactory cues fully mediated the relationship between alpha connectivity and intrusive re-experiencing symptoms, such that the correlation decreased significant and became nonsignificant following the inclusion of trauma memory scores. This memory-related mediation contrasts to emotional response, which was not associated with alpha activity (and so not included in the mediation analysis). The path strengths are indicated by beta coefficients. The parenthetical beta coefficient reflects the direct path strength after controlling for trauma memory. *p < 0.05.

To elucidate the memory mechanism through which attenuated alpha activity was related to greater intrusive re-experiencing symptoms, a mediation analysis was performed between alpha posterior→frontal GC and intrusive re-experiencing symptoms, with olfactory trauma memory (combat and non-combat collapsed) serving as the mediator. Bias-corrected bootstrap mediation analysis revealed a full mediation by olfactory trauma memory (indirect effect: *β* = −0.16, SE = 0.11, 95% CI = [−0.500, −0.011]), such that weakened alpha connectivity increased re-experiencing symptoms through heightened trauma memory (for both non-combat and combat cues; Fig. 5).

### Supplemental Analyses

A multiple regression was performed on intrusive re-experiencing symptoms with trauma memory for and emotional response to odors (combat and non-combat) as regressors to test whether the association between intrusive re-experiencing symptoms and trauma memory for olfactory cues was over and beyond emotional response. The overall model was significant (*F*_2, 34_ = 5.47, *p* = 0.007), and there was a unique association between intrusive re-experiencing and trauma memory (*sr* = 0.45, *p* = 0.007) over and beyond emotional response (*sr* = 0.12, *p* = 0.506).

An additional sensitivity analysis using multiple regression was performed with all PTSD symptom clusters as predictors of trauma memory for combat and non-combat odors. The overall model was marginally significant (*F*_2, 34_ = 2.49, *p* = 0.064), as qualified by a unique association between intrusive re-experiencing and trauma memory (*sr* = 0.39, *p* = 0.020) over and beyond all other PTSD symptom clusters (*p*’s > 0.182).

Similarly, all analyses were re-analyzed including either PTSD diagnosis or total PTSD symptom severity (PCL Total score) as a covariate to determine if the above effects were modulated by PTSD diagnosis of overall PTSD symptom severity. There was no effect of PTSD diagnosis (*p*’s > 0.81) or total PTSD symptom severity (*p*’s > 0.47) on any of the reported results, akin to the unique association with intrusive re-experiencing symptomatology.

Alternative mediation models were performed, which ruled out the other two variables (vs. trauma memory) as possible mediators of the association across alpha connectivity, trauma memory, and intrusive re-experiencing symptoms. Specifically, alpha connectivity was not related to trauma memory through intrusive re-experiencing symptoms (indirect effect: *β* = 1.32, SE = 1.32, 95% CI = [−0.362, 5.309]). Similarly, intrusive re-experiencing symptoms were not related to trauma memory through alpha connectivity (indirect effect: *β* = 1.32, SE = 1.44, 95% CI = [−0.483, 5.558]).

## Discussion

Intrusive re-experiencing reflects the involuntary recall or re-living of traumatic events characterized by vivid, sensory-rich details. In a sample of combat veterans, we demonstrated an association between heightened intrusive re-experiencing symptoms and attenuated resting-state alpha posterior→frontal connectivity. As resting-state alpha posterior→frontal connectivity represents intrinsic inhibition of sensory propagation to higher-order regions, this result suggests that impairments in intrinsic sensory inhibition can contribute to intrusive re-experiencing symptoms. We also observed that exaggerated olfactory trauma memory not only correlated with intrusive symptom severity and alpha connectivity (beyond olfactory emotional response), it also mediated their association. Taken together, the current findings highlight a specific sensory anomaly (i.e., intrinsic sensory disinhibition), which over-activates trauma memory and thereby triggers intrusive trauma re-experiencing in combat veterans.

Dominant models of intrusive re-experiencing symptoms (and PTSD in general) have largely focused on excessive threat response (e.g., detection and appraisal) and deficient emotion regulation, whereby trauma memory and intrusive re-experiencing arise via heightened threat processing and attenuated executive control^2–4,69^. Indeed, we observed a significant relation between exaggerated emotional response to combat odors and intrusive symptoms. However, pitting emotional response against trauma memory for the odors (as a factor of Response in the ANOVAs reported above), we observed that intrusive re-experiencing symptoms were particularly associated with exaggerated trauma memory (accounting for 25% of the variance). This selective association was further corroborated by a confirmatory multiple regression analysis with both emotional response and trauma memory as regressors, indicating a unique relation between trauma memory and intrusive symptoms over and beyond emotional response.

Sensitivity analyses further showed no association between trauma memory and other PTSD symptom clusters (i.e., avoidance of trauma reminders, negative alterations in mood and cognition, and hyperarousal), highlighting the inherent role of trauma memory in intrusive re-experiencing. Our mediation analysis assessing the relation between trauma memory, intrusive re-experiencing, and alpha connectivity considered alternative models and ruled out intrusive re-experiencing or alpha connectivity as the mediator among these variables, further accentuating the mediating role of trauma memory. Finally, we observed no modulatory effect of PTSD diagnosis or overall PTSD symptom severity on associations among these variables, emphasizing the specificity of the association between sensory disinhibition and trauma memory to intrusive re-experiencing symptoms.

The exaggerated trauma memory in combat veterans with more severe intrusive symptoms was found to extend to both combat and non-combat odors. Indicative of its link to PTSD pathology, this false recognition for non-combat odors echoes false memories of non-trauma cues commonly observed in individuals with PTSD^70,71^. It also aligns with clinical manifestations of intrusive memories, which are associated with a wide range of cues, including those inherently neutral and bearing little association with trauma^72^. That said, extensive evidence exists in support of heightened or preferential processing of threatening or trauma-related stimuli (across auditory, visual, and somatosensory modalities) in PTSD^73–77^. As such, we surmise that this association of neutral, non-combat odor trauma memory with intrusive symptoms may suggest a general (emotionally non-specific) memory pathway to intrusive symptoms (as suggested by the sensitivity analysis showing a unique association between olfactory trauma memory and re-experiencing, over and beyond the other symptom clusters). This notion is consistent with the dual representation theory of trauma memory in that simple (non-threat) sensory cues alone would suffice to activate sensory representations of trauma memory and elicit intrusive re-experiencing^11,12^. In addition, this association could reflect the unique intimacy between olfaction and emotion (and memory)^78–80^, such that even neutral odors can be inherently emotional and capable of activating trauma memory, especially in individuals with elevated symptoms of intrusions.

Our observation of attenuated posterior→frontal alpha connectivity in combat veterans with greater symptoms of intrusive re-experiencing augments the growing evidence of sensory disinhibition in PTSD^17,18,21^. As mentioned above, alpha oscillations serve a critical role in sensory gating and inhibition^29,81^. Specifically, the bottom-up posterior→frontal propagation of alpha activity can mediate communications from the sensory cortex to the frontal lobe, inhibiting unwanted sensory information flow (e.g., sensory distracters) to higher-order processing^30^. This functional inhibition actively supports prioritized perceptual and cognitive functions by suppressing response to irrelevant objects and distractors^23,82–85^. Alternatively, attenuated alpha activity would impair this sensory inhibition such that unwanted sensory inputs would propagate to higher-level (frontal) cortices to perturb these cognitive processes^31,83,86,87^. As our data suggest, this impaired inhibition of sensory processing in combat veterans can over-activate memory representations, eliciting memory intrusions that precipitate symptoms of trauma re-experiencing.

That this attenuation of alpha connectivity was observed during the resting state (with minimal visual input) underscores the intrinsic nature of this pathological mechanism, akin to the spontaneity of intrusive memories. Furthermore, the specific isolation of aberrant long-range alpha connectivity, as opposed to local alpha power, highlights the relevance of the directional sensory→frontal inhibition, relative to focal modulation of sensory cortical excitability, in spontaneous trauma memory activation. Importantly, this connectivity anomaly here resonates with a growing body of literature identifying aberrations in resting-state activity across global brain networks linking posterior and frontal cortical regions in PTSD^88–95^, emphasizing the importance of dysfunctional intrinsic networks in the neuropathophysiology of PTSD by disrupting various sensory, affective, and cognitive processes.

The posterior→frontal propagation of alpha oscillations is known to originate in the occipital and parietal sensory cortices to target various frontal regions, including the anterior insula and anterior cingulate cortex^30,32,33,96^. The anterior insula and anterior cingulate cortex are postulated to be key neural substrates of the sensory-based system of trauma memory^12^. They are also key hubs of the salience network, which supports salience detection and vigilant response driven by bottom-up sensory inputs^97^. Importantly, attenuated alpha activity is coupled with heightened salience network activity^98^, which are found to coalesce in PTSD^17,99,100^. As such, it stands to reason that attenuated alpha posterior→frontal connectivity combined with heightened salience network activity in trauma-exposed individuals would precipitate pathological overactivation of sensory-based trauma memory, resulting in vivid, sensory-rich intrusions of traumatic events.

While intrusive re-experiencing takes place in all sensory modalities, the visual system has been most extensively studied^11^. Nonetheless, odors are known to be strong triggers of trauma memories in PTSD^48–50,101^. Human olfaction is deeply intertwined with memory^46^, and olfactory cues are potent elicitors of involuntary memory, as famously described by Proust^79,102^. In addition, the olfactory neuroanatomy is intricately connected with the insula and anterior cingulate cortex^78,103^, which would receive intensified bottom-up input from the primary olfactory cortex in an anxious state^104^. Consistent with these ideas, our finding of olfactory trauma memory mediating the association between sensory disinhibition and intrusive trauma re-experiencing implies that fleeting whiffs of odorous air in the environment may inadvertently trigger strong trauma memories and intrusive symptoms.

The present findings should be interpreted with a number of caveats. Notably, the size of the subsample (*N* = 35) was modest. While the main effects were medium to large in size (accounting for 16–25% of variance) and survived correction for multiple comparisons, statistical power may have been limited for more subtle effects. Additionally, the use of more advanced methods, such as magnetoencephalography (MEG) or simultaneous EEG-functional magnetic resonance imaging (EEG-fMRI), would provide more insights into the neural mechanism involved in this sensory-based model of intrusive re-experiencing. Finally, while the use of a homogenous trauma-exposed sample (i.e. combat veterans) allowed for specific and controlled testing of combat-related processing, future research in other trauma-exposed samples is needed to extend this model to the general PTSD population.

Negative intrusions are observed in multiple psychiatric disorders. However, the strong perceptual vividness and rich, concrete sensory features of intrusive re-experiencing in PTSD sets these symptoms apart from abstract intrusive thoughts that are common in depression and other anxiety disorders^11^. This sensory-rich quality would render the intrusions highly experiential and, to a great extent, perceived as occurring in the present. This distinct nature of trauma-related intrusions strongly implicates sensory aberrations and promotes sensory-based conceptualization of this disorder. Towards that end, our findings complement current models emphasizing top-down regulation and inhibition^2,5^, promoting a mechanistic, sensory account of PTSD pathology. Namely, deficits in bottom-up sensory inhibition allow for mundane sensory inputs from the environment to spontaneously activate sensory representations of trauma memories, evoking involuntary recall and re-experiencing of traumatic events.

## Supplementary information

Supplemental Information.

## Data Availability

Data generated from the current study are available from the corresponding author on reasonable request.
