# Supplementary material for: Intrinsic sensory disinhibition contributes to intrusive re-experiencing in combat veterans
Source: Sci Rep. 2020 Jan 22;10:936. doi: 10.1038/s41598-020-57963-2 (PMC6976606; doi:10.1038/s41598-020-57963-2)
Supplement: Supplementary file 1 — Supplemental Information. [file 41598_2020_57963_MOESM1_ESM.docx]

**Intrinsic sensory disinhibition contributes to intrusive re-experiencing in combat veterans**

**Supplemental Information**

Kevin J. Clancy, M.S., Alejandro Albizu, B.S., Norman B. Schmidt, Ph.D., & Wen Li, Ph.D.

Department of Psychology, Florida State University, Tallahassee, FL

**SUPPLEMENTAL INFORMATION**

**SUPPLEMENTAL METHODS**

*FASTER* *Algorithm*

The *Fully Automated Statistical Thresholding for EEG artifact Rejection* (FASTER) algorithm was used for the detection and removal of artifacts within the data. Using a z-score threshold of ± 3, the FASTER algorithm detects and corrects for artifacts within single channels, individual epochs, independent components, and within-epoch channels. FASTER first interpolates deviant channels from the continuous data using the EEGLAB spherical spline interpolation function. Data were then segmented into non-overlapping, 1-second long, mean-centered epochs. Epochs were then rejected based on z-scores ± 3 within parameters of amplitude range, variance, and deviation. Epoched data were then re-referenced to averaged mastoids and submitted to independent component analysis (ICA) decomposition using the Infomax algorithm. Artefactual components (i.e. muscular artifacts, eye blinks and saccades, electrode “pop-offs”, etc.) were automatically detected and removed from the data. Lastly, deviant channels within individual, cleaned epochs were interpolated again using the EEGLAB spherical spline interpolation function.

**SUPPLEMENTAL RESULTS**

*No association between alpha power and olfactory memory or emotional response*

An omnibus rANCOVA (Category x Response x alpha power) of olfactory response revealed no main effect of alpha power on ratings (*p* = .359) or interactions with Category or Response (*p*’s > .280).

*Trauma memory for non-combat odors associated with intrusive re-experiencing symptoms over and beyond combat odors*

A multiple regression was performed entering trauma memory for combat and non-combat odors as separate regressors on intrusive re-experiencing symptoms to assess their unique roles in intrusive re-experiencing symptoms. The overall model was significant (*F*_2, 34_ = 6.20, *p* = .005), with a unique association between intrusive re-experiencing and trauma memory for non-combat odors (*sr* = .36, *p* = .036) over and beyond combat odors (*sr* = .05, *p* = .776).

*Frontal🡪posterior alpha connectivity was not related to intrusive re-experiencing symptoms*

To demonstrate the specificity of the posterior🡪frontal direction of alpha connectivity on intrusive re-experiencing symptoms, multiple regressions were performed using frontal 🡪 posterior alpha connectivity. Neither left (*sr* = -0.07, *p* = .537) nor right (*sr* = -0.12, *p* = .264) were associated with intrusive re-experiencing symptoms. Figure 1 further demonstrates the dominance of the posterior🡪frontal direction of alpha connectivity at rest, relative to frontal🡪posterior, substantiating its integral role in regulating resting state networks responsible for the orchestration of sensory and cognitive processes.
